# microRNA-125b-5p is a promising novel plasma biomarker for alveolar echinococcosis in patients from the southern province of Qinghai

**DOI:** 10.1186/s12879-021-05940-z

**Published:** 2021-03-07

**Authors:** Cao Deping, Jiang Bofan, Zhang Yaogang, Pang Mingquan

**Affiliations:** 1 Department of Human Parasitology, Guilin Medical College, Guilin, 541101 Guangxi Zhuang Autonomous China; 2 The Department of Pathogenic Biology of Qinghai University Medical College, Xining, 810001 Qinghai Province China; 3 The Echinococcosis Key Laboratory of Affiliated Hospital of Qinghai University, Xining, 810001 Qinghai Province China

**Keywords:** *Echinococcus multilocularis*, Alveolar echinococcosis, Biomarker, hsa-miR-125b-5p, Plasma

## Abstract

**Background:**

Alveolar echinococcosis (AE) is caused by parasitic infection by *Echinococcus multilocularis*. Its diagnosis is usually based on clinical symptoms, ultrasound, and other imaging methods. MicroRNAs (miRNAs) play important roles in disease processes and can exist in a highly stable cell-free form in body fluids. It is important to identify specific, sensitive diagnostic markers for early diagnosis and evaluation of AE. In this study, we examined hsa-miR-125b-5p as a potential plasma biomarker of *E. multilocularis* infection.

**Methods:**

Plasma samples from patients with AE and healthy individuals were screened for the presence of five miRNAs using miRNA chips. We used quantitative polymerase chain reaction to measure miRNA expression levels in plasma and liver tissue samples from patients with AE.

**Results:**

hsa-miR-125b-5p was stably upregulated in the plasma and liver tissue samples from patients with AE.

**Conclusions:**

The results suggest that hsa-miR-125b-5p may be a promising biomarker for early, non-invasive diagnosis of AE.

## Background

*Echinococcus multilocularis* is a small tapeworm belonging to the family Taeniidae of the class Cestoda. The parasite requires two different hosts to complete its lifecycle. Adult worms live in the intestine of carnivorous definitive hosts, such as foxes or wolves. The worms produce a large number of eggs that are released into the environment via the feces of the host. These eggs are ingested from contaminated food or drink by intermediate hosts, such as ruminants, rodents, or humans [1–3]. The eggs develop into larvae that grow into cysts called alveolar echinococcosis (AE) in the liver of the intermediate hosts, encysting and producing large numbers of protoscoleces, thus causing the zoonotic parasitic disease. When canines ingest infected rodents or viscera from intermediate hosts, the protoscoleces in the AE cysts in the canine intestine are activated and develop into adult worms in the intestine, completing the life cycle.

AE is particularly common in areas with developed animal husbandry, such as the northwest and Tibetan plateau in China, especially the Qinghai Tibetan plateau [4]. AE resembles a slow-growing liver cancer (called “worm carcinoma”), progressively infiltrating neighboring tissues and organs and even metastasizing to the brain, lungs, and other organs, thereby causing serious complications [5]. AE thus poses a significant threat to human health [6]. When left untreated, its five- and 10-year mortality rates are 52 and 96%, respectively [7]. AE poses a significant burden wherever it occurs [8]. AE is diagnosed primarily based on medical history, clinical findings, imaging techniques, and the detection of specific antibodies [9].

These diagnostic methods have drawbacks, and there is no stable and quantifiable indicator for evaluating therapeutic effects or follow-up in AE [10]. Recently, it was reported that circulating microRNAs (miRNAs) could serve as biomarkers for the detection of parasitic infections [11–13]. miRNAs are short, endogenous, non-coding RNAs that post-transcriptionally regulate gene expression by binding to the 3′-untranslated regions of target mRNAs [14]. Several studies have demonstrated the feasibility of stable detection of circulating miRNAs in the serum and plasma. miRNAs have been used as biomarkers for the early diagnosis, classification, and prognosis of various tumors, liver damages, and other diseases [15–20]. For example, sja-miR-2b-5p and sja-miR-2c-5p have been used as biomarkers with high specificity and sensitivity for the diagnosis of *Schistosoma japonicum* infection, while miR-277 and miR-3479-3p have been used to detect *Schistosoma mansoni* infection [21]. A group of miRNAs, including miR-125b, are significantly dysregulated in the plasma of patients with malaria involving multiple organ failure, indicating that miRNAs could be used as biomarkers for severe malaria infection [22]. Similarly, miR-125b-5p has been found to enhance the diagnostic potential of carcinoembryonic antigens for early-stage colon cancer [23].

miRNA assays have high sensitivity and specificity and can detect miRNAs in a small amount of blood, making sampling minimally invasive. The use of miRNAs in mouse sera as diagnostic biomarkers for AE has been studied [24], but there have been few studies on the use of miRNAs for the diagnosis of AE. The aim of this study was to validate the upregulation of miR-125b-5p in plasma after infection with *E. multilocularis* using quantitative polymerase chain reaction (qPCR).

## Methods

### MicroRNA expression profiling and detection

Twenty subjects (10 AE cases and 10 healthy cases) were randomly selected from AE inpatients and healthy outpatient individuals at the Affiliated Hospital of Qinghai University. This study was approved by the ethics review board of the Affiliated Hospital of Qinghai University (approval number: P-SL-2019054) for miRNA chip screening. An mParaflo miRNA microarray assay was performed by an external service provider (LC Sciences, Houston, TX, USA). Hybridization images were collected using a laser scanner (GenePix 4000B, Molecular Devices, San Jose, CA, USA) and digitized using Array-Pro image analysis software (Media Cybernetics Inc., Rockville, MD, USA). Data were analyzed by first subtracting the background and then normalizing the signals using a locally weighted regression filter. For two-color experiments, the ratio of the two sets of detection signals (log2 transformed, balanced) was calculated, and significant differences were determined using t-tests. Significantly different signals were defined as those with *P*-values less than 0.05. Multi-array normalization and clustering analysis were performed using a hierarchical method with average linkage and Euclidean distance metric. Clustering plots were generated for miRNAs with a total signal density of more than 1000 using the MultiExperiment Viewer software (v4.0, 2006) from the Institute for Genomic Research. The different miRNAs are shown in supplementary materials (Table 1).

**Table 1:** The prediction of target genes of miRNAs

miRNA name	Log2 (G2/G1)	*P*-value	Target Sequence (5′ to 3′)	Accession No.
hsa-miR-6849-3p	−7.96	0.015	ACCAGCCUGUGUCCACCUCCAG	MIMAT0027599
hsa-miR-3165	−3.93	0.047	AGGUGGAUGCAAUGUGACCUCA	MIMAT0015039
hsa-miR-377-5p	−2.96	0.046	AGAGGUUGCCCUUGGUGAAUUC	MIMAT0004689
hsa-miR-4725-3p	−1.97	0.035	UGGGGAAGGCGUCAGUGUCGGG	MIMAT0019844
hsa-miR-937-5p	−7.42	0.001	GUGAGUCAGGGUGGGGCUGG	MIMAT0022938
hsa-miR-203a-3p	6.88	0.003	GUGAAAUGUUUAGGACCACUAG	MIMAT0000264
hsa-miR-577	3.11	0.005	UAGAUAAAAUAUUGGUACCUG	MIMAT0003242
hsa-miR-7113-3p	6.38	0.006	CCUCCCUGCCCGCCUCUCUGCAG	MIMAT0028124
hsa-miR-125b-5p	2.42	0.0126	UCCCUGAGACCCUAACUUGUGA	MIMAT0000423
hsa-miR-6738-3p	3.20	0.0143	CUUCUGCCUGCAUUCUACUCCCAG	MIMAT0027378
hsa-miR-32-5p	5.09	0.0153	UAUUGCACAUUACUAAGUUGCA	MIMAT0000090
hsa-miR-938	−2.01	0.0881	UGCCCUUAAAGGUGAACCCAGU	MIMAT0004981
hsa-let-7e-5p	−3.07	0.0182	UGAGGUAGGAGGUUGUAUAGUU	MIMAT0000066
hsa-miR-935	−5.77	0.0251	CCAGUUACCGCUUCCGCUACCGC	MIMAT0004978
hsa-miR-382-5p	−3.91	0.056	GAAGUUGUUCGUGGUGGAUUCG	MIMAT0000737
hsa-miR-520e	−1.49	0.00623	AAAGUGCUUCCUUUUUGAGGG	MIMAT0002825

### Plasma from patients with AE and healthy individuals

We collected plasma from 44 inpatients with AE (22 males and 22 females) and 44 healthy outpatient individuals (22 males and 22 females) at the Affiliated Hospital of Qinghai University. This study was approved by the Ethics Review Board of the Affiliated Hospital of Qinghai University (approval number: P-SL-2019054). All patients were diagnosed based on their clinical symptoms and imaging data. Whole blood samples were collected in tubes, allowed to clot at 4 °C for 2 h, and centrifuged at 12,000×*g* for 10 min at 4 °C. Plasma was separated from the whole blood samples, and centrifuged at 12,000×*g* for 10 min at 4 °C to remove cell debris and blood platelets. Hemolyzed samples were excluded. Subsequently, the supernatant was transferred to fresh tubes and stored at − 80 °C until further analysis.

### Collection of liver tissues from patients with AE and healthy individuals

AE lesions and sections distant from the lesion (3 × 3 × 3 mm^3^) were resected from the liver, washed three times in phosphate-buffered saline, and stored in liquid nitrogen until further analysis.

### Cell culture and transfection

L-O2 hepatocytes were cultured in Dulbecco’s modified Eagle’s medium supplemented with 10% fetal bovine serum and penicillin/streptomycin at 37 °C in a humidified atmosphere containing 5% CO_2_. Approximately 50 nM of hsa-miR-125b-5p or control inhibitor, both purchased from JIKAI (Shanghai Jikai, China), was transfected into L-O2 cells for 48 h using riboFECTCP transfection kits (Guangzhou RiboBio Co., Guangzhou, China), according to the manufacturer’s instructions. All experiments were performed using cells in the log phase.

### RNA extraction

Total RNA was isolated from plasma and tissue samples using TRIzol, according to the manufacturer’s instructions. The concentration and integrity of isolated RNAs were evaluated using an Agilent Bioanalyzer 2100 (Agilent Technologies, Santa Clara, CA, USA). cDNA was synthesized using PrimeScript Reverse Transcriptase (TIANGEN Biotech, Beijing, Co. Ltd., Beijing, China) according to the manufacturer’s protocol. Primers targeting hsa-miR-125b-5p (reverse transcription (RT): 5′-UCCCUGAGACCCUAACUUGUGA-3′; PCR forward primer: 5′-TCCCTGAGACCCTAACTTGTGA-3′; and PCR reverse primer: 5′-GTGCAGGGTCCGAGGT-3′) and U6 (RT primer: 5′-UGAGGUAGGAGGUUGUAUAGUU-3′; PCR forward primer: 5′-TGAGGTAGGAGGTTGTATAGTT-3′; and PCR reverse primer: 5′-GTGCAGGGTCCGAGGT − 3′) were purchased from Sangon Biotech (Shanghai) Co., Ltd. (Shanghai, China).

### qPCR

qPCR was performed using a LightCycler® 480II and Tiangen SYBR Green PCR Kits. U6 was used as the endogenous control for miRNA amplification. The reaction volume was 25 μL, and was set up according to the TB GreenTM Premix Ex TaqTMII protocol. All samples and blanks were analyzed in triplicate. The reaction conditions were as follows: pre-denaturation at 95 °C for 30 s, followed by 95 °C for 5 s and 60 °C for 30 s for 40 cycles. The melting curves were generated using the following temperatures: 95 °C for 5 s and 60 °C for 1 min. Relative miRNA expression level was calculated using the 2^-△△Ct^ method and normalized to U6 levels.

### Inhibition of hsa-miR-125b-5p in treated L-O2 hepatocytes

L-O2 hepatocytes were transfected with lentiviral vector (LV), LV-hsa-miR-125b-5p-inhibitor, or the negative control virus CON137 and incubated for 5 days. The transfected cells were subsequently analyzed with the Cell Counting Kit-8 (Dojindo, CK04, Japan) for 2 h according to manufacturer’s instructions to determine the absorption at 450 nm in a time-dependent manner. The resulting OD_450_ represents the number of viable cells.

### Fluorescence-activated cell sorter analysis for apoptosis

LV-hsa-miR-125b-5p-inhibitor transfection solution and L-O2 hepatocytes were cultured in six-well culture plates in triplicate. To ensure that a detectable number of cells were present, a cell density of ≥5 × 10^5^ cells/mL was used. Confluence was 85% on the fifth day after transfection. The cells were collected and centrifuged at 250×*g* for 5 min at 4 °C, washed with cold PBS, resuspended in complete medium, and centrifuged at 250×*g* for 3 min. Cells were resuspended in 200 μL 1× binding buffer, and 10 μL Annexin V-APC was added to stain the cells. The cells were incubated for 10–15 min at room temperature (20–25 °C) in the dark, and subsequently analyzed using BD Cytoflow.

### Statistical analysis

Data are presented as the mean ± standard deviation. Independent sample *t-*tests were performed to identify significant differences between the groups, using GraphPad Prism 8 (https://www.graphpad.com/scientific-software/prism/#prism-8-new). *P* < 0.05 was considered to indicate a significant difference.

## Results

### microRNA expression in plasma samples

Using a cutoff of *P* < 0.01 and |log2foldchange| ≥ 2 [13], we chose to investigate the expression of five miRNAs (has-miR-4725-5p, has-miR-125b-5p, hsa-miR-520e, has-miR-3165, and let7e-5p) in the plasma of patients with AE. The miRNAs in black font in Table 1 are those confirmed by qRT-PCR. Only has-miR-125b-5p was stably upregulated in the plasma of AE patients. hsa-miR-520e was not detected in plasma from patients with AE. Although miR-4725-5p, has-miR-3165, and let7e-5p were also detected, their expression was unstable in plasma from AE patients.

### qPCR analysis of hsa-miR-125b-5p expression

**Fig. 1:**
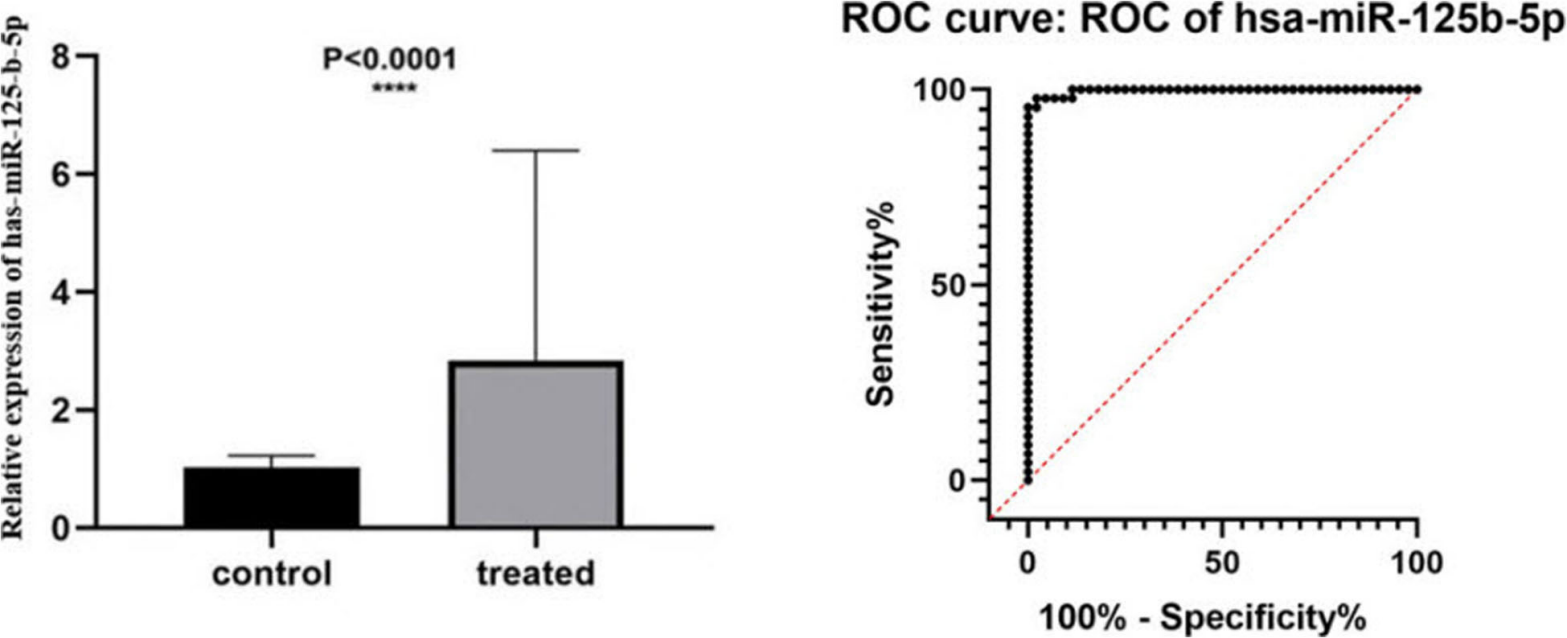
Relative expression of hsa-miR-125b-5p in the plasma of patients with AE (treated) and healthy individual (control). The receiver operating characteristic curve for the relative expression of hsa-miR-125b-5p in healthy individual and patient with AE plasma

**Fig. 2:**
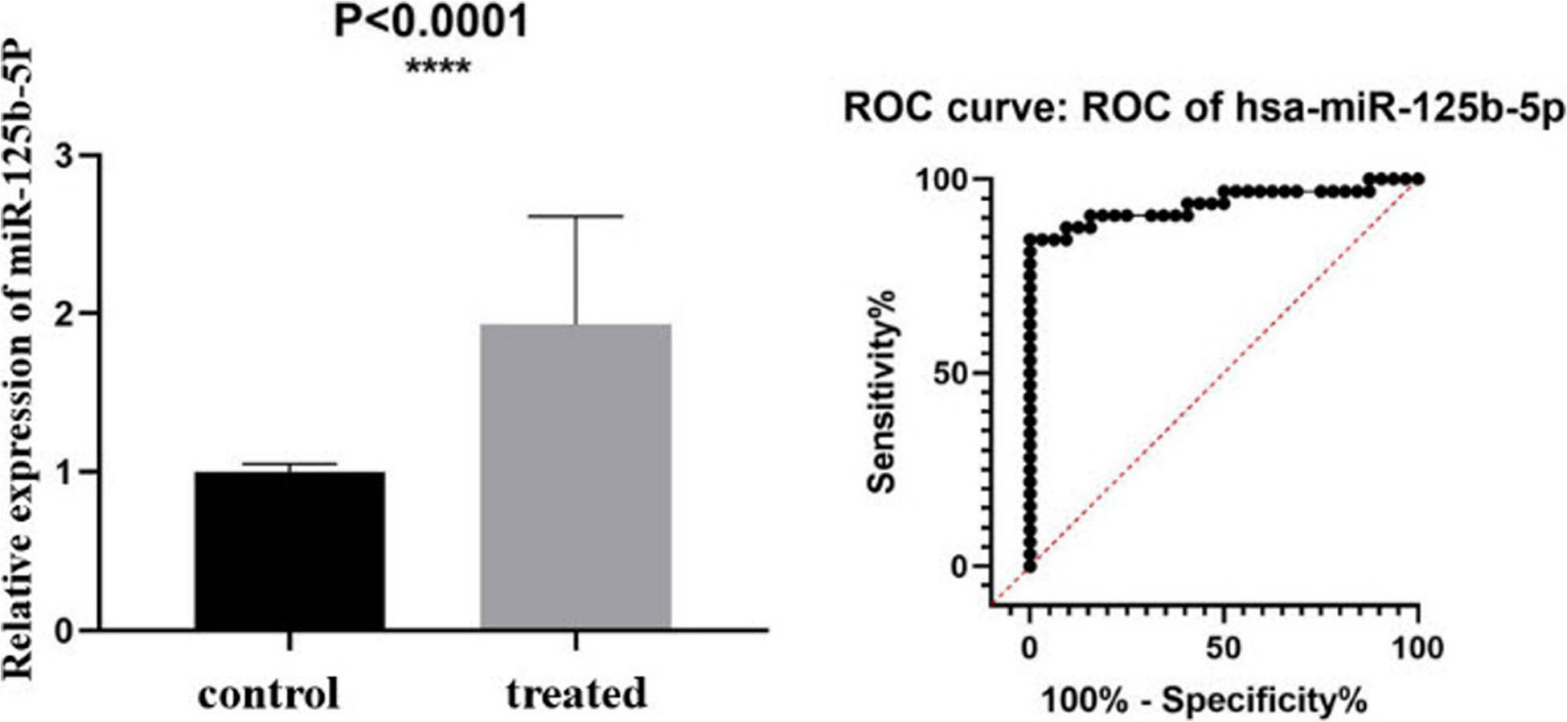
Relative expression of hsa-miR-125b-5p in AE lesions (treated) and sections distant from the lesion of liver (control). The receiver operating characteristic curve for the relative expression of hsa-miR-125b-5p in AE lesions and sections distant from the lesion of liver

qRT-PCR showed that hsa-miR-125b-5p was stably upregulated in the plasma samples of patients with AE (*P* < 0.001; Fig. 1). The area under the receiver operating characteristic (ROC) curve calculated for miR-125b-5p was 0.9967 (95% confidence interval [CI]: 0.9907–1.000). q-PCR showed that the expression of hsa-miR-125b-5p in 8 liver lesions from patients with AE and healthy individuals was statistically significant (*P* < 0.001; Fig. 2). The area under the ROC curve was 0.9365 (95% CI: 0.8701–1.000).

### Inhibition of hsa-miR-125b-5p in treated L-O2 hepatocytes

**Fig. 3:**
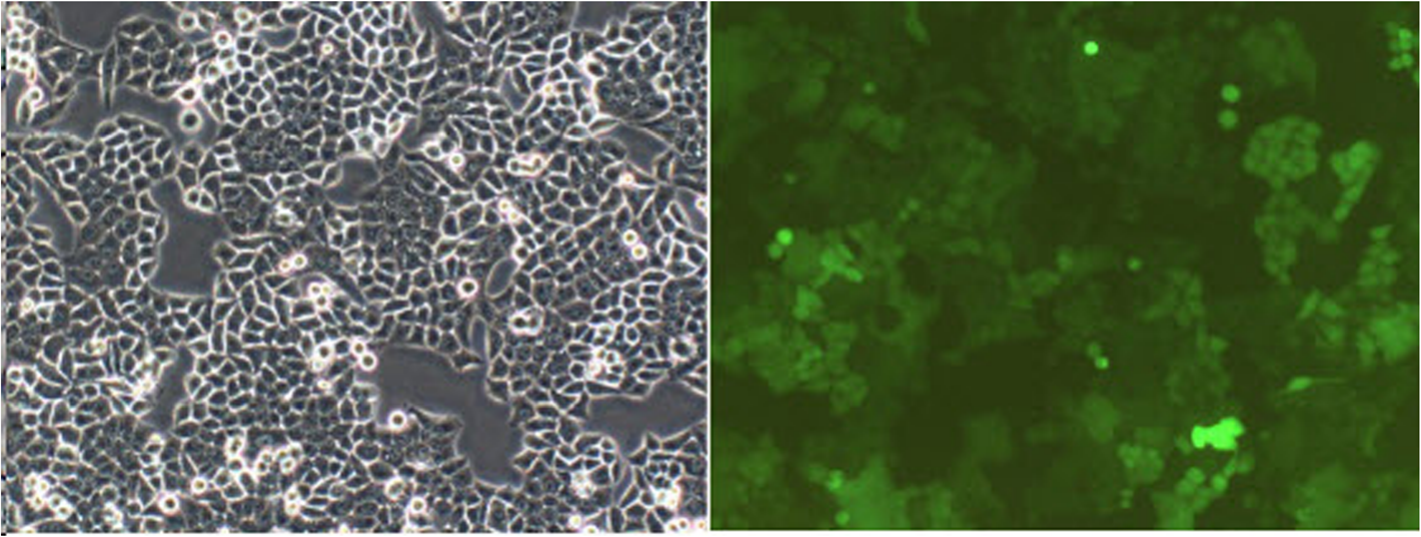
LV-hsa-miR-125b-5p inhibitor-transfected normal L-O2 hepatocytes (magnification, 100×)

**Fig. 4:**
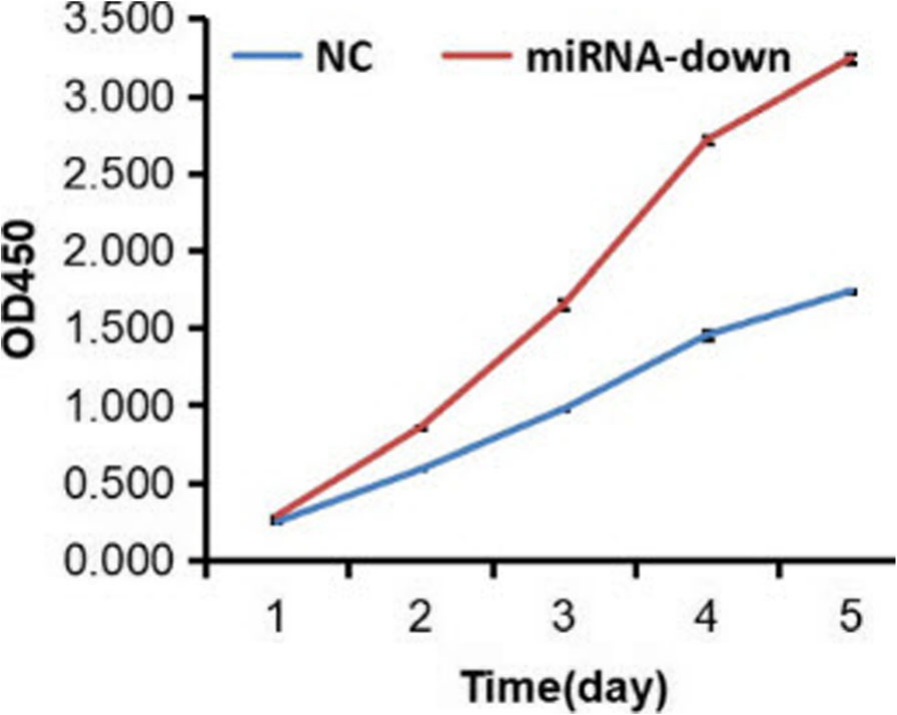
*t*-test analysis of cell proliferation of LV-hsa-miR-125b-5p inhibitor-transfected and control cells (NC; *P* < 0.05)

Normal L-O2 hepatocytes were transfected with LV, LV-hsa-miR-125b-5p-inhibitor, or negative control virus CON137 (Fig. 3). On the fifth day after treatment, the OD_450_ of the LV-hsa-miR-125b-5p-inhibitor group was 3.242 ± 0.032, whereas that of the control group was 1.738 ± 0.008 (*P* < 0.05; Fig. 4).

**Fig. 5:**
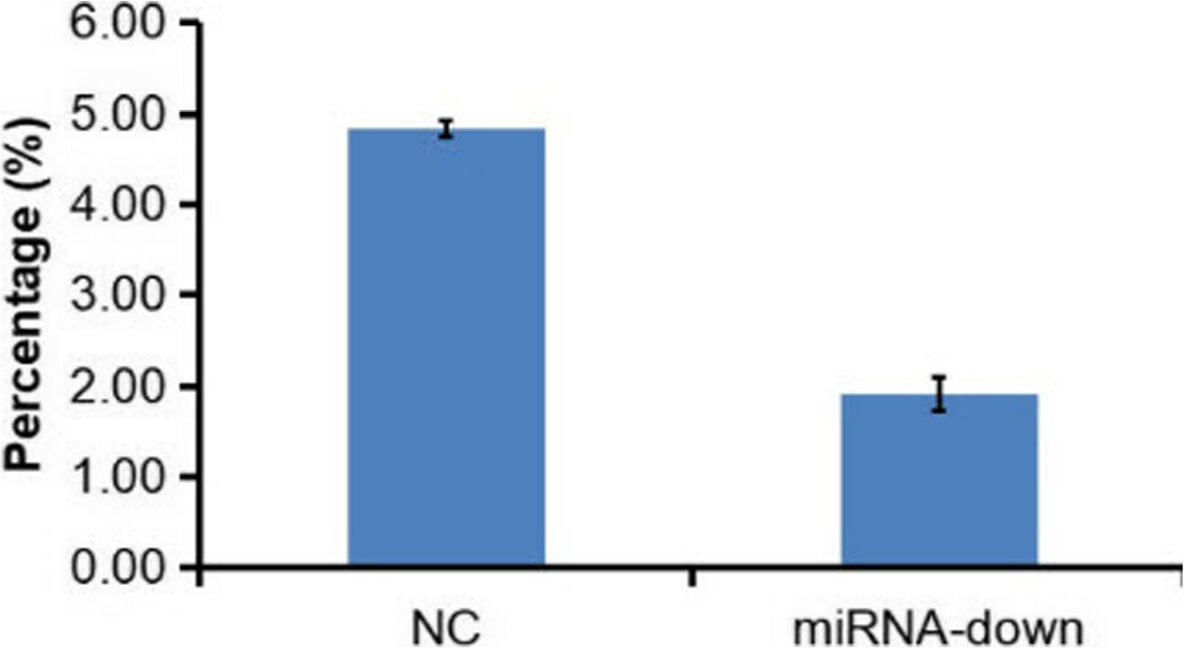
*t*-test analysis of apoptosis in control and LV-hsa-miR-125b-5p inhibitor-transfected L-O2 cells (*P* < 0.05)

The apoptosis rate (%) of the LV-hsa-miR-125b-5p-inhibitor group was 4.84 ± 0.09, whereas that of the control group was 1.91 ± 0.18 (*P* < 0.05). Apoptosis was decreased between the control and LV-hsa-miR-125b-5p inhibitor-transfected L-O2 cells after 5 days (*P* < 0.05) (Fig. 5).

## Discussion

miRNAs are small, non-coding regulatory RNAs that can play important roles in the pathogenesis of parasitic diseases [25–27]. miRNAs are stably expressed not only in tissues, but also in blood or body fluids. Recent reports have shown that these extracellular RNAs are associated with a variety of liver diseases, including viral hepatitis, steatohepatitis (both alcoholic and nonalcoholic), and drug-induced liver injury [28]. Further, miRNAs are considered biomarkers for early diagnosis of diseases, including liver injury [29]. Obtaining plasma for miRNA detection is non-invasive and less traumatic than collecting tissues for the same purpose. Moreover, plasma miRNAs can be stably detected and thus have a great research prospect. Further, serum miRNA expression has been widely studied in liver lesions. The expression of miR-222, miR-21, and miR-223 in the sera of patients with chronic hepatitis B-induced hepatocellular carcinoma was significantly different from that of the normal control [30]. Cai et al. [31] reported that miR-122, miR-21, and miR-34a can be used as biomarkers to support the early diagnosis of schistosomiasis. Jia et al. [32] found that the expression of mmu-miR-712-3p, mmu-miR-511-5p, and mmu-miR-217-5p was significantly increased species-specifically in the plasma of mice infected with *Toxoplasma gondii*, suggesting that miRNAs can be used as biomarkers for *T. gondii* infection. These evidences suggest that abnormal miRNA expression can indicate the progress of disease.

miR-125b-5p, a member of the miR-125 family, was found to regulate the proliferation of differentiated tumor cells, as first reported in 2005 by Lee et al. [33]. Studies on miRNA-125b-5p in liver tumors and liver microbial diseases showed that the expression of miRNA-125b-5p in the plasma and tissue of patients is significantly altered [34–36]. Recent studies have also explored the possibility of using plasma miRNA-125b-5p as a diagnostic biomarker for early-stage cervical cancer and rheumatoid arthritis [37, 38].

In this study, the expression level and diagnostic value of hsa-miR-125b-5p were validated by q-PCR in the plasma and liver lesions of patients with AE compared with those of healthy individuals. Transfection with LV-hsa-miR-125b-5p-inhibitor reduced apoptosis in L-O2 hepatocytes in vitro. We thus speculated that miR-125b-5p plays a role in AE and that inhibiting miR-125b-5p may protect hepatocytes and reduce AE progression. It is not clear why miRNA-125b-5p expression increases in the plasma and liver lesions of patients with AE. Therefore, further study is required to elucidate the mechanism underlying the increase in miRNA-125b-5p expression in the plasma/sera and liver lesions of patients with AE after liver injury. In this study, miR-125b-5p exhibited an increasing trend. Our results can be used as the basis for further research to determine whether miR-125b-5p is really valuable in the diagnosis and prognosis of AE.

## Conclusions

The detection of plasma miRNAs is non-invasive, and can be applied for the investigation, prevention, and diagnosis of diseases. From the results of this study, miR-125b-5p might serve as a promising diagnostic marker for AE.

## Data Availability

The datasets used in this study are available from the corresponding author on reasonable request.

## Abbreviations

AE: Alveolar echinococcosis
miRNAs: Circulating microRNAs
qPCR: Quantitative polymerase chain reaction
LV: Lentiviral vector
ROC: Receiver operating characteristic
95% CI: 95% confidence interval
